# Effect of vaccination of cattle with the low virulence Nc-Spain 1H isolate of *Neospora caninum* against a heterologous challenge in early and mid-gestation

**DOI:** 10.1186/1297-9716-44-106

**Published:** 2013-11-01

**Authors:** Silvia Rojo-Montejo, Esther Collantes-Fernández, Francisco Pérez-Zaballos, Sonia Rodríguez-Marcos, Javier Blanco-Murcia, Antonio Rodríguez-Bertos, Antoni Prenafeta, Luis Miguel Ortega-Mora

**Affiliations:** 1 SALUVET, Animal Health Department, Faculty of Veterinary Sciences, Complutense University of Madrid, Avda. Puerta de Hierro s/n, 28040 Madrid, Spain; 2 HIPRA, Avda. La Selva, 17170 Amer, Spain

## Abstract

Live vaccines have emerged as one of the most potentially cost-effective measures for the control of bovine neosporosis. Previous studies have shown that Nc-Spain 1H is a naturally attenuated isolate of *Neospora caninum* and that immunisation with live Nc-Spain 1H tachyzoites generated a protective immune response in mice. The aim of this study was to evaluate the safety and efficacy of immunisation in cattle. *N. caninum*-seronegative heifers were immunised subcutaneously twice with 10^7^ live Nc-Spain 1H tachyzoites prior to artificial insemination. No adverse reactions or negative effects on reproductive parameters were recorded following immunisation. In immunised and non-challenged heifers, no foetal deaths were observed, and none of the calves was congenitally infected. The efficacy against *N. caninum*-associated foetal death and vertical transmission was determined after challenge with high doses of the Nc-1 isolate at 70 and 135 days of gestation, respectively. After the challenge in early gestation, the immunisation induced a protection of 50% against foetal death. In addition, the microsatellite analysis performed in PCR-positive tissue samples from foetuses that died after challenge infection showed that the profiles corresponded to the challenge isolate Nc-1. A degree of protection against vertical transmission was observed after challenge at mid-gestation; calves from immunised heifers showed significantly lower pre-colostral *Neospora*-specific antibody titres than calves from the non-immunised/challenge group (*P* < 0.05). Strong antibody and interferon gamma responses were induced in the immunised heifers. This study indicates that the immunisation before pregnancy with the Nc-Spain 1H vaccine isolate appeared to be safe and reduced the occurrence of *N. caninum*-associated abortion and vertical transmission in experimentally infected cattle. In light of these encouraging results, the next step for testing this live attenuated candidate should be the assessment of its efficacy and safety in naturally infected cattle.

## Introduction

Bovine neosporosis is one of the major parasitic diseases causing reproductive failure in cattle worldwide. Unfortunately, there are no drugs available to prevent *N. caninum*-associated abortion and vertical transmission, and control of this disease relies on effective management. In this context, a previous economic model recommended vaccination as the most cost-efficient alternative, even when an only partially effective vaccine was employed [1,2]. While several approaches have been developed to obtain vaccines against *N. caninum*, those methods based on attenuated isolates appear to be the most promising candidates for feasible live vaccines [3,4].

Recently, a new *N. caninum* isolate (Nc-Spain 1H) was obtained from the brain of a clinically healthy but congenitally infected calf and demonstrated to have reduced virulence by means of *in vitro* and *in vivo* experiments. The Nc-Spain 1H isolate displayed lower invasion and proliferation efficiency in cell culture than did the Nc-1 isolate [5,6]. The pathogenicity of Nc-Spain 1H was examined in BALB/c mice; in a dose-titration experiment, this isolate failed to induce clinical signs of infection or mortality, and no parasites were detected in the brains of these mice during the chronic stage, even in those given doses of 10^7^ tachyzoites. In a pregnant mouse model, the offspring survival rate from Nc-Spain 1H-infected dams was almost 100%, and *N. caninum* was detected in only one pup [5]. Furthermore, the inoculation of Nc-Spain 1H tachyzoites in cattle at 70 days of gestation did not induce foetal death [7]. A recent study also proved that this isolate was highly efficacious in preventing congenital and cerebral neosporosis in mice [8]. These data indicate that Nc-Spain 1H is a low-virulence isolate, and, consequently, may be a safe candidate for live-vaccine development in cattle.

Therefore, the aim of this study was to evaluate the safety and efficacy of the Nc-Spain1H isolate at preventing foetal death and vertical transmission in cattle after a heterologous challenge at early and mid-gestation.

## Materials and methods

### Ethical statement

All of the protocols involving animals were approved by the Animal Research Committee of the Complutense University, Madrid, Spain in compliance with the proceedings described in the Regulation of Internal Regime for Animal Research Committee (published at BOUC, no. 2, at 9 February 2006) and the EU legislation (Council Directive 86/609/EEC).

### Parasites

Live tachyzoites from the *N. caninum* Nc-Spain 1H isolate [5] and from the Nc-1 isolate [9] were used for the immunisation and for the heterologous challenge, respectively. Both isolates were maintained *in vitro* by continuous passage in MARC-145 cell monolayers as previously described [10] to ensure healthy and actively dividing parasites. To preserve its original biological properties, the Nc-Spain 1H isolate was subjected to a limited number of culture passages (passage no. 9–17). The Nc-1 isolate, which had been maintained in Vero cells for long-term passages, was propagated under new culture conditions using MARC-145 cells. This shift from Vero cells to a new cell line was expected to minimise potential changes in the biological characteristics of the parasite due to prolonged cell culture maintenance [10]. The Nc-1 tachyzoites were inoculated in cattle after a limited number of culture passages in MARC-145 cells (passage no. 15). Parasite viability and numbers were determined by trypan blue exclusion followed by three aliquot countings in a Neubauer chamber. The infection dose was adjusted with phosphate-buffered saline (PBS) to the required doses for immunisation or challenge in a final volume of 5 mL per heifer. The parasites were administered to cattle within 2 h of harvesting from the tissue culture.

Nc-1 tachyzoites used for DNA extraction and as antigens for specific IFNγ detection and ELISA techniques were washed three times in sterile PBS (pH 7.4). Host cell debris was separated by first passing the mixture through a 25-gauge needle then through PD-10 columns (GE Healthcare Life Sciences, Uppsala, Sweden) [11]. Cell-free Nc-1 tachyzoites were pelleted by centrifugation (600 × *g*, 10 min) and frozen at -80 °C until use. To obtain *N. caninum* soluble protein antigens, purified tachyzoites were suspended in 1 mL of 10 mM Tris–HCl containing 2 mM phenylmethylsulphonyl fluoride (Sigma, St. Louis, MO, USA) and were disrupted by sonication (Sonifier 450, Branson Ultrasonic, Danbury, CT, USA) in an ice bath. Cell debris and unlysed cells were removed by centrifugation (10 000 × *g*, 20 min, 4 °C). The protein concentration of the supernatant was quantified using the Micro BCA protein assay (Pierce, Rockford, IL, USA), and the supernatant was aliquoted and frozen at -80 °C.

### Animals and experimental design

Forty-eight Holstein Friesian heifers aged 16–24 months and negative for *Brucella abortus*, *Leptospira* spp., Infectious Bovine Rhinotracheitis Virus (IBRV) and Bovine Viral Diarrhoea Virus (BVDV) were selected from the same dairy farm. Heifers were tested twice by ELISA (CIVTEST BOVIS NEOSPORA, HIPRA, Girona, Spain) for evidence of *N. caninum* exposure, and were all found to be seronegative. Before starting the experiment, the heifers were vaccinated against BVDV and IBRV (HIPRABOVIS**®** 4, HIPRA, Girona, Spain).

While at the dairy farm, 26 heifers were randomly selected and subcutaneously (sc) immunised twice at 4-week intervals with 10^7^ tachyzoites of the Nc-Spain 1H isolate in PBS over the left prescapular lymph node prior to artificial insemination; the remaining heifers received PBS. All of the animals were synchronised to be in oestrus at the time of the booster immunisation. Synchronisation was accomplished by administering two intramuscular injections of a synthetic PGF_2α_ analogue (Prosolvin, MDS Animal Health, Salamanca, Spain) at 11-day intervals. At 72 h after the last injection, the animals were artificially inseminated (AI) with semen from a *N. caninum*-seronegative bull. Booster immunisation was conducted at the time of the AI. Pregnancy was confirmed by ultrasonography in 31 heifers on days 35 and 57 after AI. After the diagnosis of pregnancy, 27 immunised and non-immunised pregnant heifers were moved to an experimental farm at Complutense University of Madrid (UCM).

Animals maintained in experimental conditions were allocated into 6 experimental groups (groups 1–4 with 5 animals, group 5 with 4 animals and group 6 with 3 animals) as described in Table 1. Groups 1, 2 and 4 were sc-immunised with the Nc-Spain 1H isolate prior to AI. The protection against foetal death was evaluated in groups 2 and 3 after intravenous (iv) challenge with 10^7^ live Nc-1 tachyzoites at 70 days of gestation. The efficacy against vertical transmission was examined in groups 4 and 5, where animals were iv-challenged with 4 × 10^8^ Nc-1 tachyzoites at 135 days of gestation. Groups of immunised/non-challenged (group 1) and non-immunised/non-challenged (group 6) animals received an inoculum of PBS. The remaining animals were maintained under field conditions at the dairy farm and divided into two groups consisting of immunised heifers (group 7, *n* = 11) and non-immunised heifers that received PBS (group 8, *n* = 10) (Table 1). These animals became pregnant during the study following the routine reproductive program in operation on the farm. None of the animals in these groups was challenged.

**Table 1:** Summary of groups

**Group**^**a**^	**Immunisation**	**Challenge time**	**Challenge dose**	**Number of animals**
	**(Nc-Spain 1H tachyzoites)**^**b**^	**(days of gestation)**	**(Nc-1)**	
1 (Immunised/non-challenged)	10^7^	-	-	5
2 (Immunised/70-challenged)	10^7^	70	10^7^	5
3 (Non-immunised/70-challenged)	None	70	10^7^	5
4 (Immunised/135-challenged)	10^7^	135	4 × 10^8^	5
5 (Non-immunised/135-challenged)	None	135	4 × 10^8^	4
6 (Non-immunised/non-challenged)	None	-	-	3
7 (Immunised/non-challenged)	10^7^	-	-	11
8 (Non-immunised/non-challenged)	None	-	-	10

^a^ Groups 1 to 6 were maintained at the experimental farm (UCM), whereas groups 7 and 8 were kept at the dairy farm throughout the experiment.

^b^ Heifers were subcutaneously immunised twice at 4-week intervals prior to artificial insemination.

### Clinical monitoring and sample collection

The safety of the vaccine isolate (Nc-Spain 1H) was determined by daily monitoring of the health status of immunised heifers (groups 1, 2, 4 and 7) in order to detect possible adverse reactions. Rectal temperatures were recorded daily for a week after the immunisation and booster. Animals with temperatures above 39.5 °C were considered febrile. The ability of the Nc-Spain 1H isolate to induce foetal death or to be vertically transmitted was investigated in immunised/non-challenged heifers located at the experimental farm (groups 1) and the dairy farm (group 7). In these groups, foetal viability, detection of pre-colostral *Neospora*-specific antibodies in calves and the presence of parasite DNA and lesions in tissues from offspring were measured. Additionally, microsatellite analysis was used to discriminate the challenge isolates (Nc-1) from the immunisation isolates (Nc-Spain 1H) in nested-PCR *N. caninum*-positive tissue samples from immunised/challenged animals (groups 2 and 4) after challenge. The impact of the immunisation on the reproductive performance was also studied by comparing the following reproductive parameters between immunised (groups 1, 2, 4 and 7) and non-immunised heifers (groups 3, 5, 6 and 8): number of AI per conception, fertility at first AI, age at first AI and age at first calving.

The protective efficacy of the immunisation with live Nc-Spain 1H tachyzoites against foetal death and vertical transmission was determined by comparing immunised and non-immunised heifers after challenge at early (group 2 vs. group 3) and mid-gestation (group 4 vs. group 5), respectively. In these groups, rectal temperatures were measured daily for a week after challenge and then twice weekly until the end of the experiment. Foetal viability was monitored during gestation; if foetal death occurred, the presence of parasite DNA and lesions in foetal tissues were determined. Vertical transmission was identified in calves after birth by the presence of *Neospora*-specific antibodies in pre-colostral blood and of parasite DNA and lesions in tissue samples.

In the cattle maintained under experimental conditions (groups 1–6), foetal growth and viability was monitored by trans-rectal ultrasonography weekly following challenge for 45 days then every two weeks until the end of the pregnancy. When ultrasound examinations detected a non-viable foetus, the animal was examined 24 h later; if the foetal death was confirmed, the dam was sacrificed with an intravenous overdose of embutramide and mebezonium iodide (T-61®, MDS Animal Health, Salamanca, Spain). The remaining heifers were monitored until delivery and slaughtered 1 week after. In cattle kept under field conditions, foetal viability was evaluated following the routine reproductive programme in operation in the dairy farm. Pregnancy status was determined on days 40–45 and 210–215 of gestation; between these two tests, the walking activity of the animal was monitored by pedometers to assess the possibility of reproductive failure. After calving, these heifers remained on the farm and were used for dairy production. All of the calves from cattle kept under both experimental and field conditions that were born at full-term were euthanised within 1 day of birth by an intravenous overdose of embutramide and mebezonium iodide (T-61®, MDS Animal Health, Salamanca, Spain). Pre-colostral blood samples from calves were collected after calving in plain evacuated tubes for serology analysis. Foetuses, calves and heifers were necropsied immediately after euthanasia and tissues were recovered aseptically. Brain, heart and liver samples from all of the foetuses and calves (groups 1–8) and brain and placenta samples from the dams maintained under the experimental conditions (group 1–6) were placed in 10% formol saline or stored at -80 °C until use for histopathology or for PCR and microsatellite analyses, respectively.

Blood samples from heifers were collected after immunisation every two weeks during the experiment by coccygeal venipuncture in plain evacuated and heparinised tubes for serology and IFNγ analysis, respectively. After the pregnancy diagnosis, blood samples from heifers that continued on to the dairy farm were collected in plain evacuated tubes for serology every 1–3 months from immunisation for 22 months.

### DNA extraction and PCR

Three different areas from each of the above-mentioned tissues from foetuses, calves and dams were randomly selected and pooled. Subsequently, 5–8 g of the pool were homogenised in sterile PBS (dilution 1:2) in a stomacher (“Masticator” IUL, Barcelona, Spain) for 2 to 5 min. Placentome samples were minced using a sterile scalpel. DNA was extracted from aliquots of 50 μL of the homogenised tissues (3 for foetuses and 12 for calves) or three placental tissue samples (15 mg of tissue per sample) using the Real Pure Genomic DNA Extraction Kit (Durviz, Valencia, Spain) following the manufacturer’s instructions. *N. caninum* DNA was obtained from 10^7^ tachyzoites. The concentration of DNA was determined by spectrophotometry and adjusted to 50 ng/μL. A total of 5 μL were used for PCR amplification.

Nested PCR of the internal transcribed spacer region of *N. caninum* was carried out with four oligonucleotides as described by Buxton et al. [12]. A secondary amplification product was visualised by 1.8% agarose gel electrophoresis and ethidium bromide staining. DNA equivalent to 10^2^ tachyzoites was used as the positive PCR control. To avoid false positive reactions, DNA extraction, PCR sample preparation and electrophoresis were performed in separate rooms with different sets of instruments, aerosol barrier tips and disposable gloves. Moreover, negative control samples were included in each set of DNA extractions and PCR reactions.

### Microsatellites analysis for isolate identification

Approximately 250 ng of DNA extracted from PCR-positive tissue samples from foetuses, calves and dams was used as the template for the amplification by nested PCR of 13 microsatellite markers as previously described [13,14]. For automated allele sizing, all reverse primers in the secondary PCR were fluorescently end-labelled. Amplified products were prepared with HiDi formamide and Gene Scan-500 (LIZ) Size Standards (Applied Biosystems, CA, USA). The size of the fluorescent PCR product was determined using a 48-capillary 3730 DNA analyser (Applied Biosystems, CA, USA) and analysed with GeneMapper® V 3.5 Software [14].

### Histopathological examination

A histopathological study was carried out on different sections of the brain and placenta from dams and of the brain, heart and liver from foetuses and calves using routine histological methods. Tissues were fixed in 10% neutral buffered formalin and dehydrated through graded alcohols before being embedded in paraffin wax and stained with haematoxylin and eosin. The analysis was based on the observation of lesions according to previous descriptions [15-17], and lesions were classified as none detected/unrelated (-), consistent with (+), or characteristic of (++) bovine neosporosis.

### *N. caninum*-specific antibody responses

Blood samples from heifers and calves were allowed to clot before centrifugation at 1500 × *g* for 10 min; the serum was removed, aliquoted and stored at -20 °C until required.

Serum samples from heifers were assayed for specific IgG antibodies using an *N. caninum* soluble extract antigen-based ELISA as previously described [18]. Serum samples were diluted to 1:100 for testing. The anti-bovine IgG1 and IgG2 monoclonal antibodies (mAbs) (Laboratorie Service International, France) were diluted to 1:4000. Serum samples were analysed in duplicate, and the mean value of the optical density (OD) was converted into a relative index per cent (RIPC) using the following formula: RIPC = (OD_405_ sample - OD_405_ negative control)/(OD_405_ positive control - OD_405_ negative control) × 100. A RIPC value ≥ 8.2 indicates a positive result.

*N. caninum*-specific IgG antibodies from pre-colostral blood from calves were measured by IFAT as described by Trees et al. [19]. *N. caninum*-specific antibodies were measured in pre-colostral serum samples using a dilution of 1:25 to the endpoint titre [18]. Complete peripheral fluorescence of tachyzoites was considered a positive result, and fluorescence was visualised with a Nikon microscope (mod. HB-10101AF; mercury lamp).

### *N. caninum-*specific IFNγ responses

Heparinised blood samples were maintained at room temperature and cultured within 2 h of collection from cattle with 100 μL of PBS (unstimulated control), concanavalin A (10 μg/mL; Sigma) to ensure the ability of the cells to respond to stimulation and secrete IFNγ, and with *N. caninum* Nc-1 isolate soluble antigen (1 μg/mL) as described previously [7,20]. To assess IFNγ production, duplicate plasma samples were tested using a commercial ELISA kit (Bovigam IFNγ kit, CSL, Australia) as recommended by the manufacturer and using positive and negative controls provided with the kit. The results are expressed as OD values.

### Statistical analysis

Prior to challenge, rectal temperatures, IgG and IFNγ responses were compared between immunised (groups 1, 2, 4 and 7) and non-immunised (groups 3, 5, 6 and 8) heifers using the repeated measures Student’s *t*-test. After challenge, differences in these parameters among all the groups were analysed individually using the repeated measures one-way ANOVA test. When statistically significant differences were found, a Duncan Multiple Range test was applied to examine all possible pairwise comparisons.

Pre-colostral antibody titres and reproductive parameters between immunised and non-immunised groups were compared using Student’s *t*-test. Lesion severity was analysed by the Mann–Whitney *U*-test. A *p-*value of less than 0.05 was considered statistically significant.

## Results

### Rectal temperature

**Figure 1:**
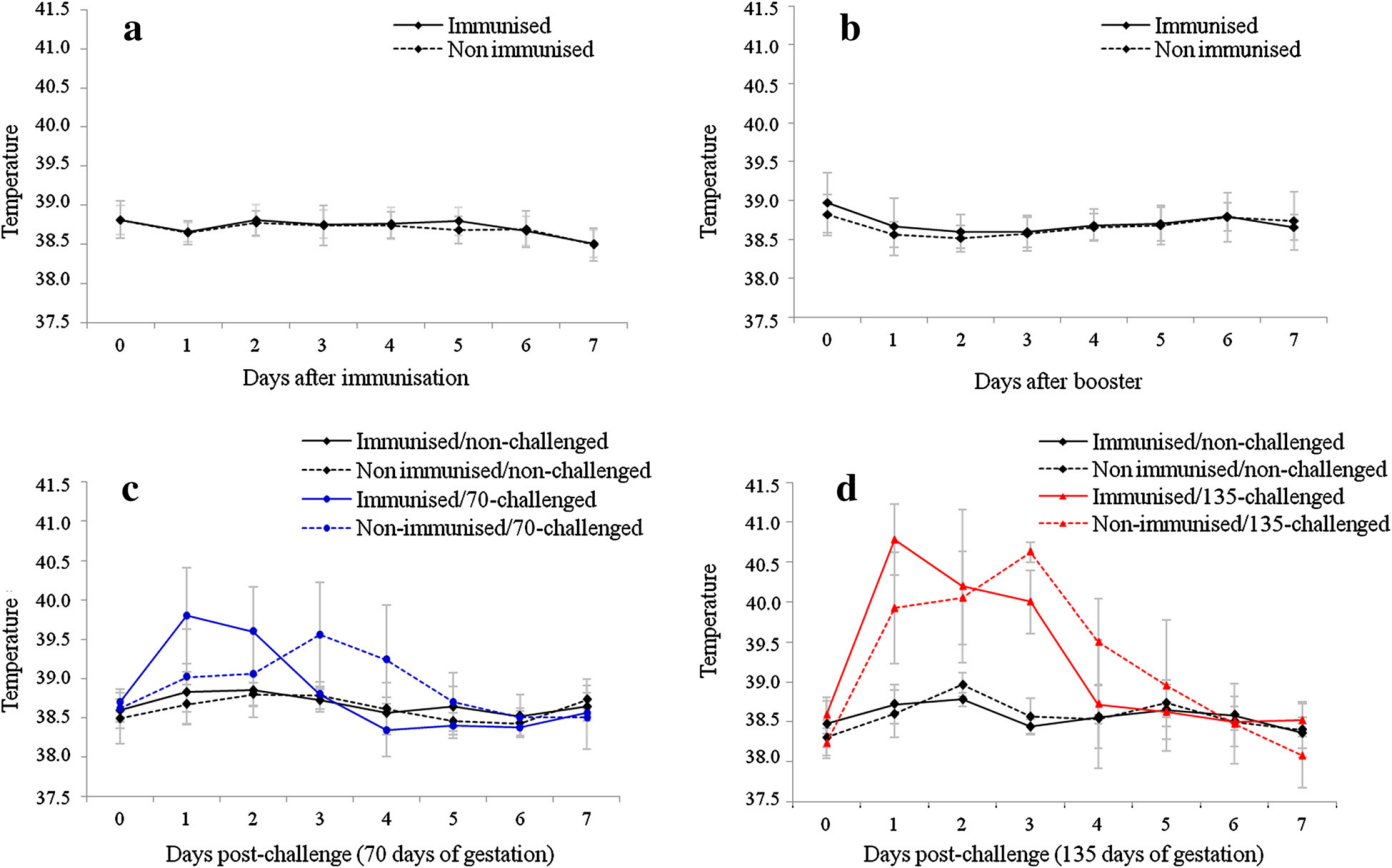
**Mean rectal temperature of cattle.** The rectal temperatures were recorded daily for a week following immunisation **(a)** and booster **(b)** with the Nc-Spain 1H isolate, and following challenge with the Nc-1 isolate in early **(c)** and mid-gestation **(d)**. Data are expressed as the mean rectal temperature (°C), and error bars represent the standard deviations for each group.

After immunisation and administration of the booster, none of the immunised heifers developed hyperthermia (≥ 39.5 °C). Although slightly higher temperatures were observed in immunised than in non-immunised animals, these differences were not significant (groups 1, 2, 4, 7 vs. groups 3, 5, 6, 8; *P* > 0.05; Figure 1a and b).

Following the challenge at early gestation, both challenged groups showed a significant rise in temperature in comparison with non-challenged groups (groups 2, 3 vs. groups 1, 6; *P* < 0.001-0.01; Figure 1c). The temperature response in group 2 (immunised/70-challenged) significantly increased at 1–2 days post-challenge (dpc) and peaked at 39.8 °C 1 dpc (group 2 vs. groups 1, 3, 6; *P* < 0.001-0.01). In group 3 (non-immunised/70-challenged), mean temperatures rose on 3–4 dpc and peaked at 39.6 °C 3 dpc (group 3 vs. groups 1, 2, 6; *P* < 0.001-0.01).

When heifers were challenged at mid-gestation, mean rectal temperature was higher in challenged groups than in non-challenged groups from 1 to 3 dpc (groups 4 and 5 vs. groups 1 and 6, *P* < 0.0001-0.01; Figure 1d). In group 4 (immunised/135-challenged), the peak mean temperature was 40.7 °C 1 dpc (group 4 vs. groups 1, 6; *P* < 0.0001-0.01), whereas in group 5 (non-immunised/135-challenged), the mean peak temperature was 40.6 °C 3 dpc (group 5 vs. groups 1, 4, 6; *P* < 0.0001-0.01).

In contrast to the temperature evolutions that occurred after immunisation, the challenge infection stimulated a febrile response that was detected earlier in immunised animals than in non-immunised animals and that had a higher magnitude after challenge at 135 days of gestation.

### Efficacy of live Nc-Spain 1H immunisation against foetal death at early gestation

When the efficacy against foetal death was evaluated after challenge early in gestation, a protection of 50% was observed in group 2 (immunised/70-challenged). In this group, foetal death was detected in 2 of the 5 heifers at 26 and 97 dpc, whereas in group 3 (non-immunised/70-challenged), foetal death occurred in 4 of the 5 animals, two at 26 dpc and the others at 77 and 83 dpc. In both challenged groups, foetal death was associated with the presence of *N. caninum* DNA and severe histopathological changes compatible with *N. caninum* infection in either foetal or placenta tissues (Table 2). In the dead foetuses, lesions mainly consisted of multifocal hepatocellular necrosis in the liver, lymphocytic myocarditis, foci of mononuclear meningoencephalitis and perivascular cuffing in brain. In placental tissue samples from dams that experienced foetal death, severe placentitis consisting of serum leakage between maternal and foetal tissues, non-suppurative inflammatory infiltrates in both the maternal and foetal mesenchyme and areas of haemorrhage and necrosis in the caruncular septa were detected. When lesion severity was compared between groups 2 and 3, the differences were found to be non-significant (*P* > 0.05).

**Table 2:** Detection of parasite DNA and histopathological changes in foetal and maternal tissues of immunised and non-immunised cattle after challenge infection with the Nc-1 isolate in early gestation

**Group**	**Time of foetal death (dpc)**^**a**^	**Foetal**	**Serology**	**Tissues from foetus/calf**									**Maternal placenta**		
		**crown-rump length (cm)**	**in progeny (IFAT)**^**c**^	**Brain**			**Liver**			**Heart**					
				**HP**^**d**^	**DNA**^**e**^	**MS**^**f**^	**HP**^**d**^	**DNA**^**e**^	**MS**^**f**^	**HP**^**d**^	**DNA**^**e**^	**MS**^**f**^	**HP**^**d**^	**DNA**^**e**^	**MS**^**f**^
2 (Immunised/70-challenged)	26	14	-	+	3/3	Nc-1	++	3/3	Nc-1	++	3/3	Nc-1	++	1/3	Nc-1
	97^b^	15	nd	+	0/3	-	++	0/3	-	++	0/3	-	++	1/3	na
	Alive	nd	-	-	0/12	-	-	0/3	-	-	0/3	-	-	0/3	-
	Alive	nd	-	-	0/12	-	-	0/3	-	-	0/3	-	-	0/3	-
	Alive	nd	-	-	0/12	-	-	0/3	-	-	0/3	-	-	0/3	-
3 (Non-immunised/70-challenged)	26	17	-	+	1/9	na	-	0/3	-	-	0/3	-	-	0/3	-
	26	16	-	+	3/3	Nc-1	++	3/3	Nc-1	++	3/3	Nc-1	++	2/3	na
	77	18	1:32	*	1/3	na	*	0/3	-	*	1/3	na	++	2/3	na
	83	30	-	*	1/3	na	++	2/3	na	++	2/2	na	++	2/3	na
	Alive	nd	-	-	0/12	-	-	0/3	-	-	0/3	-	-	0/3	-

^a^ Day post-challenge when foetal death was detected by transrectal ultrasonography. The remaining foetuses lived until the end of the experiment.

^b^ This foetus was mummified but probably died two months before (around 33 dpc) as indicated by its foetal crown-rump length. The poor quality of the samples from this mummified foetus most likely made the detection of parasite DNA unfeasible. Foetal abdominal fluid for serological analysis could not be collected.

^c^ Mean IFAT IgG antibody titres in foetal body fluids and in pre-colostral serum collected after birth in calves born alive.

^d^ Histopathological lesion severity: none detected (-), consistent with (+), and characteristic of (++) *N. caninum* infection; * Autolysed.

^e^ Fractions represent number of samples positive by nested PCR/number of samples examined.

^f^ Microsatellite analysis was performed using one sample per nested-PCR-positive tissue. Microsatellite markers could not be amplified from all of the PCR-positive tissues analysed (na). The microsatellite profiles of the tissues from which the microsatellite markers could be amplified corresponded to that of the challenge isolate (Nc-1).

The remaining heifers from groups 2 and 3 that did not experience foetal death gave birth to live, clinically normal calves. These calves were seronegative for pre-colostral *N. caninum*-specific antibodies, and neither parasite DNA nor pathological changes were observed in any examined tissues. In addition, none of the adult cattle showed *N. caninum* DNA or histopathological changes in brain tissue samples.

### Efficacy against vertical transmission in immunised heifers following infection at mid-gestation

After challenge at mid-gestation, one dead foetus was found in both groups 4 (immunised/135-challenged) and 5 (non-immunised/135-challenged), most likely due to the high challenge dose by iv route; the remaining foetuses survived throughout the gestation (Table 3). In the challenged groups, the foetuses died at 25 (group 4) and 47 (group 5) dpc. The presence of parasite DNA was detected in brain tissue samples from both of the dead foetuses, in liver tissue samples from the foetus in group 4 and in placental tissues from their dams. Similarly to the changes that occurred in dead foetuses after challenge at day 70 of gestation, the foetal death was associated with severe lesions in foetal and placental tissues from animals in both challenged groups that were compatible with *N. caninum* infection.

**Table 3:** Detection of parasite DNA and histopathological changes in tissue samples from foetuses, calves and heifers from immunised and non-immunised groups following challenge infection at mid-gestation

**Group**	**Time of foetal death (dpc)**^**a**^	**Serology in progeny (IFAT)**^**b**^	**Tissues from foetus/calf**								**Maternal placenta**		
			**Brain**			**Liver**			**Heart**				
			**HP**^**c**^	**DNA**^**d**^	**MS**^**e**^	**HP**^**c**^	**DNA**^**d**^	**MS**^**e**^	**HP**^**c**^	**DNA**^**d**^	**HP**^**c**^	**DNA**^**d**^	**MS**^**e**^
	25	-	*	2/3	Nc-1	++	2/3	na	+	0/3	++	1/3	na
4 (Immunised/135-challenged)	Alive	-	-	0/12	-	-	0/3	-	-	0/3	-	1/3	-
	Alive	1:100	-	0/12	-	-	0/3	-	-	0/3	-	0/3	-
	Alive	1:200	-	0/12	-	-	0/3	-	-	0/3	-	0/3	-
	Alive	1:200	-	0/12	-	-	0/3	-	-	0/3	-	0/3	-
5 (Non-immunised/135-challenged)	47	1:16	++	1/3	na	++	0/3	-	++	0/3	++	2/3	na
	Alive	1:800	-	0/12	-	-	0/3	-	-	0/3	-	0/3	-
	Alive	1:1600	-	0/12	-	-	0/3	-	-	0/3	-	0/3	-
	Alive	1:1600	-	0/12	-	-	0/3	-	-	0/3	-	0/3	-

^a^ Day post-challenge when foetal death was detected by transrectal ultrasonography. The remaining foetuses lived until the end of the experiment.

^b^ Mean IFAT IgG antibody titres in foetal body fluids and in pre-colostral serum collected after birth in calves born alive.

^c^ Histopathological lesion severity: none detected (-), consistent with (+), and characteristic of (++) *N. caninum* infection; * Autolysed.

^d^ Fractions represent number of samples positive by nested PCR/number of samples examined.

^e^ Microsatellite analysis was performed using one sample per nested-PCR-positive tissue. Microsatellite markers could not be amplified from all of the PCR-positive tissues analysed (na). The microsatellite profiles of the tissues from which the microsatellite markers could be amplified corresponded to that of the challenge isolate (Nc-1).

When the protection against vertical transmission was evaluated, most of the calves from both challenged groups were found to have precolostral *N. caninum*-specific antibodies; however, antibody titres were significantly lower in animals from the immunised/135-challenged group (group 4 vs. group 5; *P* < 0.01; Table 3). In this group, 3 out of 4 calves had pre-colostral *N. caninum*-specific antibodies detected by IFAT at titres ranging from 1:100 to 1:200. In contrast, all of the calves from the non-immunised/135-challenged group showed *N. caninum*-specific antibody titres ranging from 1:800 to 1:1600. Parasite DNA and lesions compatible with *N. caninum* were not detected in tissues from these calves, and only one placental tissue sample was positive by PCR in the immunised/135-challenged group (group 4). In addition, neither *N. caninum* DNA nor histopathological changes were found in brain tissues from dams.

### Safety of the subcutaneous immunisation with live Nc-Spain 1H tachyzoites

Following immunisation, nodules at the injection site were observed in 3 immunised heifers until 3 days after immunisation and in 2 immunised heifers 1 day after administration of the booster.

In immunised/non-challenged heifers maintained under both experimental (group 1) and field conditions (group 7), most of the calves survived to term and were born clinically normal with the exception of 2 of 11 calves from the group maintained in the dairy farm that died as a result of dystocia. Nonetheless, all the calves were seronegative for pre-colostral *Neospora*-specific antibodies. In addition, neither parasite DNA nor lesions were detected in the brain, heart and liver samples from calves or in the placenta and brain samples from their dams.

Microsatellite analysis was performed to distinguish the presence of the challenge isolate (Nc-1) from the immunisation isolate (Nc-Spain 1H) in PCR-positive tissues from foetuses that died during the study in both immunised/challenged groups (groups 2 and 4). Because of its low sensitivity [14], amplification of the microsatellite markers was not achieved for all of the nested-PCR positive samples. In group 2, this analysis was performed using the brain, heart and liver tissues from one of the two dead foetuses and the placental tissue from its dam (Table 2). In group 4, microsatellite sequences were amplified only from the brain samples of the aborted foetus (Table 3). In all these samples, only the microsatellite profile corresponding to the challenge isolate (Nc-1) was detected.

Finally, no differences between immunised and non-immunised groups in routinely evaluated reproductive parameters in dairy heifers were observed (Table 4), indicating that immunisation with Nc-Spain 1H tachyzoites prior to AI did not negatively impact reproductive performance.

**Table 4:** Effect of the immunisation with live Nc-Spain 1H tachyzoites on reproductive performance

	**Immunised and non-challenged group**	**Non-immunised and challenged group**
No. heifers bred	26	22
No. heifers conceived	25	22
No. stillbirths	2	4
No of AI per conception ^a^	2.5 ± 1	2.4 ± 1.2
Fertility at first AI (%) ^a^	61.5	68.2
Age at 1st AI ^b^	16.6 ± 2.2	17.0 ± 1.6
Age at 1st calving ^b^	27.6 ± 3.0	27.7 ± 2.5

^a^ Data obtained from the immunised heifers (groups 1, 2, 4 and 7, *n* = 26) and the non-immunised heifers (groups 3, 5, 6 and 8, *n* = 22) before challenge infection.

^b^ Data correspond to the immunised/non-challenged (groups 1 and 7, *n* = 16) and non-immunised/non-challenged (groups 6 and 8, *n* = 13) heifers.

### *Neospora caninum*-specific antibody response

**Figure 2:**
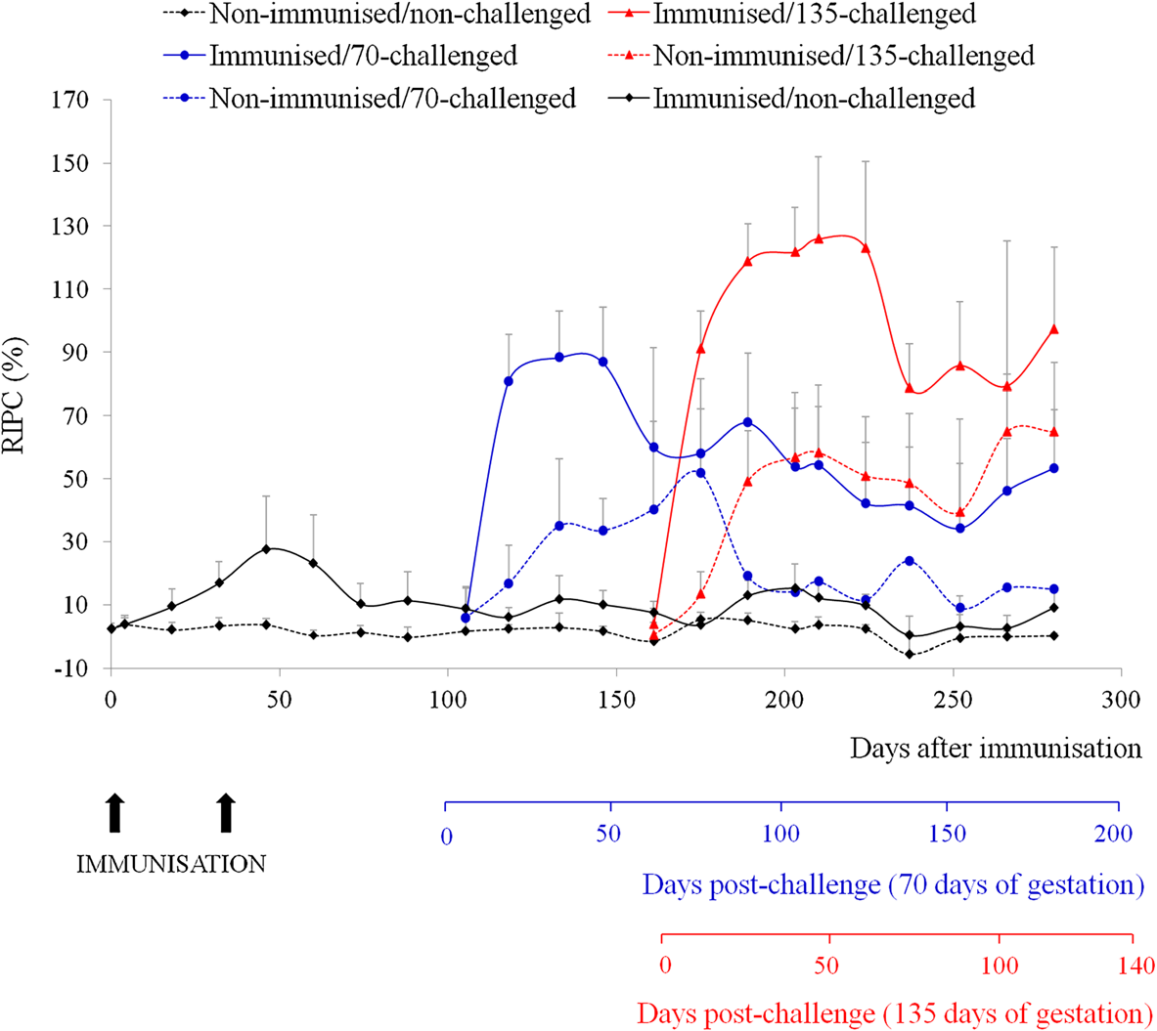
***N. caninum*-specific IgG responses in cattle kept at the experimental farm.** The IgG antibody levels were measured in heifers following immunisation with the Nc-Spain 1H isolate prior to AI and challenge with the heterologous Nc-1 isolate at early and mid-gestation. Data are expressed as the mean relative index per cent (RIPC), and error bars represent the standard deviations for each group. Only the upper standard deviation bars are shown. Positive cut-off ≥ 8.2 RIPC. The antibody concentration could not be measured in non-immunised/70-challenged group from day 77 post-challenge because most of the heifers were euthanised due to the high foetal mortality detected.

**Figure 3:**
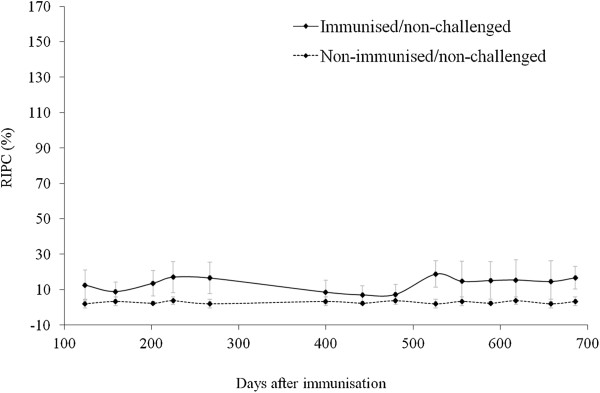
***N. caninum*-specific IgG responses in cattle maintained at the dairy farm from day 88 after immunisation.** Data are expressed as the mean relative index per cent (RIPC), and error bars represent the standard deviations for each group. Positive cut-off ≥ 8.2 RIPC.

Prior to challenge, higher antibody levels were detected between days 18 and 88 after immunisation in immunised/non-challenged heifers than in non-immunised/non-challenged heifers (group 1, 2, 4 and 7 vs. groups 3, 5, 6 and 8; *P* < 0.0001; Figure 2); antibody levels in the immunised/non-challenged heifers reached a peak at day 46 after immunisation. After day 88 post-immunisation (60 days of gestation), the antibody levels in immunised/non-challenged heifers moved to the experimental farm (group 1) decreased to levels that were close to the cut-off value (RIPC = 8.2). The IgG response of these animals remained higher than that of the non-immunised/non-challenged heifers (group 6) until the end of the experiment, with the exception of day 175, but the differences were not significant. The antibody levels in immunised/non-challenged dams kept under field conditions (group 7) fluctuated around the cut-off from day 124 until day 686 after immunisation (22 months after immunisation), and generally remained higher in these animals than in non-immunised/non-challenged animals (group 8) with the exception of days 159, 400, 442 and 480 after immunisation (*P* < 0.001; Figure 3).

Following challenge early in gestation, animals in groups 2 (immunised/70-challenged) and 3 (non-immunised/70 challenged) showed higher antibody production than those that were not challenged (Figure 2). These differences were detected in group 2 from 20 dpc onwards (group 2 vs. groups 1, 6; *P* < 0.0001-0.01), and in group 3 between 20 and 77 dpc (group 3 vs. groups 1, 6; *P* < 0.01). When both challenged groups were compared, animals in group 2 displayed higher antibody levels than animals in group 3 between 20 and 48 dpc (*P* < 0.01).

The antibody production in animals from groups challenged on day 135 of gestation (groups 4 and 5) were significantly higher than in animals from non-challenged groups (groups 1 and 6) (Figure 2). Animals from group 4 (immunised/135-challenged) showed the strongest antibody response. In this group, the antibody levels increased on 12 dpc and remained high until the end of the experiment, peaking between 26 and 61 dpc (group 4 vs. groups 1, 6; *P* < 0.0001-0.01). Heifers from group 5 (non-immunised/135-challenged) showed significantly higher levels from 26 dpc onward (group 5 vs. groups 1, 6; *P* < 0.01). When comparing both challenged groups, animals from group 4 showed significantly higher levels than animals from group 5 from 12 dpc until calving (*P* < 0.01), excluding day 103 pc.

In summary, the immunisation with Nc-Spain 1H induced a specific-IgG response that was detectable during the three months after immunisation. After challenge in early and mid-gestation, the magnitude of the antibody response was significantly higher in immunised/challenged groups than in non-immunised/challenged groups.

### IFNγ response after immunisation with live Nc-Spain 1H tachyzoites

**Figure 4:**
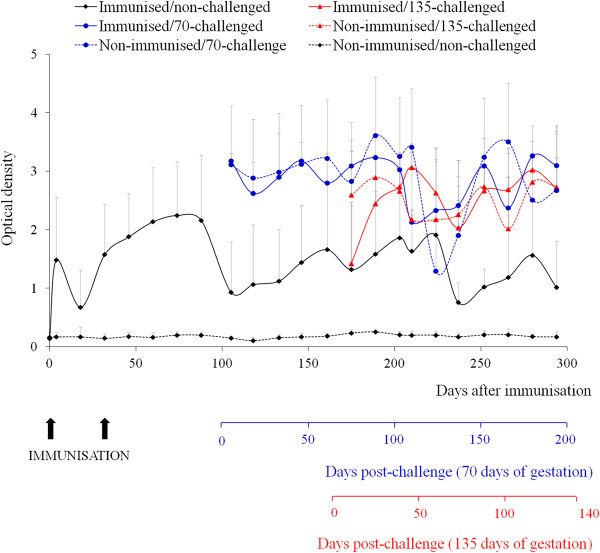
***N. caninum*-specific IFNγ response in cattle kept at the experimental farm.** The production of IFNγ in heifers after immunisation with the Nc-Spain 1H isolate prior to AI and challenge with the Nc-1 isolate is shown. Data are expressed as the mean optical density (OD), and error bars represent the standard deviations for each group. Only the upper standard deviation bars are shown.

Prior to the challenge, the antigen-specific IFNγ response in whole blood culture induced following immunisation was evaluated in immunised heifers. In these animals, IFNγ secretion was detected from day 4 after immunisation, and their levels of this cytokine remained significantly higher than those of the non-immunised heifers until the day of challenge (groups 1, 2 and 4 vs. groups 3, 5 and 6 on days 4–88 after immunisation; groups 1 and 4 vs. groups 5 and 6 on days 105–161 after immunisation; *P* < 0.001) and until the end of the study (group 1 vs. group 6 from day 175 after immunisation onwards, except days 266 and 294 after immunisation; *P* < 0.001) (Figure 4).

The IFNγ values in animals from both groups challenged early in gestation (groups 2 and 3) were high from 6 dpc until the end of the experiment (Figure 4). Animals from group 2 (immunised/70-challenged) showed higher levels than those in non-challenged groups (group 2 vs. 1 on 6–90, 138, 153 and 181 dpc; group 2 vs. group 6 from 6 dpc onward; *P* < 0.0001-0.05). In group 3 animals (non-immunised/70-challenged), a significant, specific IFNγ secretion was detected between 6 and 77 dpc compared to groups 1 and 6 (*P* < 0.05). When both challenged groups (groups 2 and 3) were compared, the differences were not significant.

After challenge at mid-gestation, strong, specific IFNγ responses were detected in animals from both challenged groups (groups 4 and 5, Figure 4). IFNγ secretion was higher in animals from the immunised/135-challenged group than in those from the non-challenged (group 4 vs. group 1, on 48 and 90–118 dpc; group 4 vs. group 6 from day 26 onwards; *P* < 0.05). A similar pattern of IFNγ secretion was detected in animals from the non-immunised/135-challenged group (group 5 vs. group 1 on 26, 48, 90 and 118 dpc; group 5 vs. group 6 from 12 dpc onwards; *P* < 0.05). When the levels of IFNγ in animals from groups 4 and 5 were compared, no significant differences were found.

These data indicate that the production of IFNγ was immediately stimulated after immunisation with live Nc-Spain 1H tachyzoites; furthermore, IFNγ levels rose significantly after challenge and remained at high levels throughout the gestation period.

## Discussion

Vaccine development for bovine neosporosis remains a high research priority because no cost-effective control measures are available. Live attenuated vaccines against other apicomplexan parasites such as *Toxoplasma* and *Eimeria* have successfully induced protective immunity by stimulation of appropriate cell mediated immune responses; a live vaccine against bovine neosporosis could effectively control *N. caninum*-associated abortions and congenital transmission. One of the most promising approaches for live vaccine development against neosporosis involves the use of naturally attenuated *N. caninum* isolates. The Nc-Nowra isolate of *N. caninum* obtained from the brain of a congenitally infected calf was previously tested as a potential live vaccine candidate in an experimental mouse model [21] and a pregnant-bovine model [3,4] and resulted in a reduction in transplacental transmission of the parasite and protection against foetal death, respectively. At the same time, the Nc-Spain 1H isolate was similarly selected as a potential vaccine candidate for its desirable biological characteristics, including low invasion and proliferation efficiency in *in vitro* assays and reduced virulence and undetectable persistence in mouse and bovine models [5,7].

Appropriate *in vitro* and animal infection models are necessary to test the safety and efficacy of a vaccine and to allow more accurate and cost effective vaccine development and candidate selection for clinical trials. In neosporosis, mouse models provide an initial proof-of-concept for different vaccine candidates. Consequently, we previously reported that the immunisation of mice with live Nc-Spain 1H tachyzoites resulted in a protective immunity that was able to efficiently control both congenital and cerebral neosporosis after challenge infection with the virulent Nc-Liv isolate [8]; these results confirm the suitability of the Nc-Spain 1H isolate as a promising vaccine candidate against bovine neosporosis.

However, accurate testing of vaccine efficacy ultimately requires trials using putative vaccine candidates in cattle as the target species. With this purpose in mind, this study evaluated whether the immunisation with live Nc-Spain 1H tachyzoites prior to mating stimulates a protective response against *N. caninum*-associated foetal death and vertical transmission using an immunisation/challenge pregnant bovine model. This animal model was based on previous bovine models of infection that efficiently reproduced the consequences of parasitic infection after challenge at early [22,23] and mid-gestation [24,25]. Indeed, these bovine models were successfully used to evaluate the efficacy of potential vaccine candidates in preventing foetal loss or vertical transmission of the parasite [3,24]. In the present work, the protective effect of Nc-Spain 1H against foetal death was primarily evaluated after iv inoculation of 10^7^ live Nc-1 tachyzoites at 70 days of gestation; a foetal mortality rate of 80% was observed in the non-immunised/challenged group. These data agree with previous works in which foetopathy was detected in all of the infected animals after iv inoculation of the dam with tachyzoites of Nc-Liv or Nc-1 isolates [22,23]. When the protection of the Nc-Spain 1H isolate against vertical transmission was evaluated, a higher challenge dose of parasites was inoculated at mid-gestation in comparison to that inoculated at early gestation. We increased the challenge dose inoculated on day 135 of gestation to 4 × 10^8^ live Nc-1 tachyzoites because infection with 10^7^ live Nc-1 tachyzoites on day 70 of gestation did not induce 100% foetal mortality in the non-immunised and challenged heifers. This challenge resulted in efficient vertical transmission of the parasite, as high *N. caninum*-specific titres were detected in all of the calves born to the non-immunised/day-135-challenged group. Previously, Innes et al. [24] described a highly valuable vertical transmission bovine model in which all calves born to dams sc-challenged at mid-gestation (day 140 of gestation) with 5 × 10^8^ live Nc-1 tachyzoites had high levels *N. caninum*-specific antibodies. However, the high challenge dose of the present study appeared to be more potent, inducing not only vertical transmission in all of the calves born at full term but also mortality in one foetus at 47 days after challenge. These differences could be due to the iv challenge route used in our study that has been reported as inducing greater parasite-associated lesions in the placenta and foetus than the subcutaneous route [23].

Safety problems are some of the main concerns and major challenges of live vaccine development. The key requirements of live attenuated vaccines against bovine neosporosis are (i) the inability of the vaccine isolate to be transmitted to the foetus, which could result in abortion or the birth of a congenitally infected animal, and (ii) the inability of the vaccine isolate to establish infection in cattle, which could result in the threat of recrudescence in subsequent pregnancies and, ultimately, the potential reversion to virulence of the parasite. Because the Nc-Spain 1H isolate was obtained from the brain of a clinically healthy but congenitally infected calf, we conducted an extensive safety evaluation of this isolate to provide sufficient assurance of its reduced virulence and persistence in cattle to justify its potential future use in field cases. To address these questions, we evaluated the ability of the Nc-Spain 1H to induce foetal death in the immunised and non-challenged heifers kept under both experimental and field conditions; in these groups, all of the foetuses were viable throughout gestation. Moreover, to rule out the involvement of the vaccine isolate in foetopathy, a microsatellite analysis, which has been used to differentiate *Neospora* isolates in previous vaccination assays [8,26], was performed in PCR-positive foetal and placental tissues samples from immunised and challenged heifers that showed foetal death. In all cases where the analysis could be performed, only the microsatellite genotype corresponding to the challenge isolate Nc-1 [13] was detected. Furthermore, all of the calves born to immunised and non-challenged heifers were seronegative at birth. This finding suggests that the Nc-Spain 1H isolate was unable to induce a detectable persistent infection in these calves. The failure to obtain evidence of *N. caninum* infection in the tissues of any of the immunised cattle may be explained by the low sensitivity of the techniques used, which were unable to detect small amounts of parasite DNA in these tissues. Nonetheless, these findings are in agreement with previously obtained data regarding the pathogenic characterisation of this isolate, in which Nc-Spain 1H neither caused foetal death nor was detected in foetal tissues or the brains of dams [7]. Altogether, these observations suggest that Nc-Spain 1H is most likely not congenitally transmitted to progeny when dams are experimentally inoculated before or during gestation.

Another important aspect of vaccine safety for bovine neosporosis is the potential negative effect of the inoculation on reproductive performance; indeed, Weber et al. [4] recently described a reduction in pregnancy rates in cattle vaccinated with live Nc-Nowra tachyzoites prior to AI, possibly as a result of an increased cytokine response that could have negatively affected embryo implantation. In the current work, heifers were sc immunised twice at 4-week intervals with live Nc-Spain 1H tachyzoites 4 weeks prior to AI. This immunisation protocol was designed based on previous evidence indicating that congenital transmission of *N. caninum* does not occur in cattle primo-infected prior to pregnancy with live tachyzoites [24]. Moreover, previous studies demonstrated that *N. caninum* infection did not interfere with the development of preimplantation stage bovine embryos [27,28]. As expected, the examination of routinely evaluated reproductive parameters proved that under the conditions of the present study, the immunisation of dairy heifers with live Nc-Spain 1H tachyzoites did not negatively affect their reproductive performance.

This study demonstrated that immunisation with live Nc-Spain 1H tachyzoites prior to pregnancy partially limited foetal death and vertical transmission. Thus, a protection against foetal death of 50% was observed in immunised heifers after challenge in early gestation. In addition, most of the calves born to immunised heifers challenged at mid-gestation were seropositive at birth, suggesting that complete protection against vertical transmission was not achieved. However, precolostral *N. caninum*-specific antibodies titres were significantly lower in animals from the immunised group than in animals from the non-immunised group, which could indicate a moderate protective response that partially controlled the number of parasites reaching placenta and, consequently, limited exposure of the calves to the parasite in utero. Previous studies using similar immunisation/challenge bovine models have shown that immunisation of cattle with live *N. caninum* tachyzoites results in higher levels of protection than those described in the present study after sc immunisation with live Nc-Spain 1H tachyzoites. On the one hand, iv vaccination with live Nc-Nowra tachyzoites completely prevented foetal loss after a virulent challenge with 10^7^ tachyzoites of the Nc-Liv isolate at 70 day of gestation [3]; similarly, a recent work on the efficacy of vaccination with live Nc-Nowra tachyzoites confirmed the high degree of protection against *Neospora*-associated foetal loss conferred by either iv or sc vaccination [4]. On the other hand, the infection of cattle with live Nc-1 tachyzoites prior to pregnancy afforded a protection of 100% against congenital transfer to the foetus after a homologous challenge in mid-gestation at day 140 [24]. Some of the biological characteristics of the *N. caninum* isolates selected as candidates for live vaccine, such as their ability to replicate and actively disseminate, could determine the parasite burden in host tissues. These factors could influence the antigenic dose to which the immunised animals are exposed and, consequently, affect the robustness of the protective immune response. In this sense, the Nc-Spain 1H isolate, which previously displayed low *in vitro* invasion and proliferation capacities [6], could slowly replicate in host tissues and provide a weak, repeated antigenic stimulus. These properties could explain the only partial efficacy induced by the Nc-Spain 1H isolate in comparison with other more prolific isolates such as Nc-1. Nonetheless, this fact favours the establishment of a suitable balance between vaccine safety and efficacy because potential problems related to residual virulence after vaccination with live tachyzoites could be mitigated with the use of an attenuated strain. Because the natural mechanism underlying the low virulence of Nc-Spain 1H is unknown and no studies regarding the recrudescence or reversion to virulence of this isolate have been conducted, further investigations should be performed in cattle to guarantee the safety of this attenuated isolate in the natural host. In addition, the primo-infection at early and mid-gestation achieved in this work by inoculating the cattle intravenously with notably high parasite doses has been deemed too aggressive for testing the efficacy of Nc-Spain 1H in a bovine model; as a result, the vaccine potency could potentially be underestimated. Accordingly, the performance of the Nc-Spain 1H-vaccine in the target population (chronically infected cattle) must be evaluated in the future.

The immunological response provides evidence of exposure to live Nc-Spain 1H tachyzoites because all immunised animals developed *N. caninum*-specific IgG and IFNγ responses after parasite inoculation. In particular, those immunised animals that were not challenged and maintained under both experimental and field conditions showed high IgG antibody levels during the three months after immunisation. Subsequently, their antibody levels decreased to levels similar to those observed in the non-immunised heifers, and they fluctuated around the cut-off value until the end of the experiment, suggesting that there was no recrudescence of the Nc-Spain 1H infection during gestation that might have led to foetal infection. A similar situation was previously described when an experimental infection of cattle with live tachyzoites of this isolate resulted in antibody levels that declined to undetectable levels from the third week after infection [7]. However, immunisation induced high levels of IFNγ production that persisted throughout the experiment. Williams et al. [3] reported a similar cellular-mediated immune response in cattle 19 weeks after they were immunised with live tachyzoites, suggesting the presence of antigen-specific memory T cells associated with protection against foetal death in the immunised animals. It has been widely reported that IFNγ plays a major role in protection against *N. caninum* infection by limiting parasite growth [29,30]. Nevertheless, because a strong, specific IFNγ response was similarly induced after challenge in both immunised and non-immunised animals, a protective immune response could not definitively be associated with IFNγ levels. Further studies are needed to investigate the immunological mechanisms involved in the protective immunity induced by the Nc-Spain 1H isolate.

In conclusion, our data suggest that immunising cattle with live tachyzoites of the Nc-Spain 1H isolate appears to be safe and generates a protective immune response that partially prevents *N. caninum*–associated abortions and the vertical transmission of this parasite. We therefore support the use of this isolate as a promising vaccine candidate for the control of bovine neosporosis. Further field studies should be conducted to address whether protective immunity could be induced by Nc-Spain 1H against neosporosis in naturally infected cattle. In addition, safety studies should be conducted to evaluate possible residual virulence or recrudescence in the target animals. Finally, taking into consideration the potential problems related to the efficacy of a live vaccine product and its short shelf-life, the development of formulations containing stabilisers that preserve the viability of tachyzoites would be valuable for commercial manufacture and distribution of a live vaccine.

## Competing interests

The authors declare that they have no competing interests.

## Authors’ contributions

ECF, AP and LMO conceived and designed the study. SiRM, ECF and LMO participated in its coordination; SiRM, ECF, JBM and FPZ immunised and challenged the dams and performed their clinical examination. SiRM, ECF, FPZ and SoRM participated in the necropsy and sampling of the animals and performed the molecular biology and serological analyses of the samples. ARB carried out the histopathological analysis of the samples. SiRM and ECF performed the statistical analyses and interpreted the results. SiRM, ECF and LMO wrote the manuscript; with inputs from all authors. All authors read and approved the final manuscript.
